# Assessment of diagnosis and treatment practices of diarrhoea in children under five in Maputo-Mozambique

**DOI:** 10.1016/j.ijans.2022.100507

**Published:** 2022

**Authors:** Nórgia Elsa Machava, Elsa Maria Salvador, Fhumulani Mulaudzi

**Affiliations:** a Department of Nursing Sciences, Faculty of Health Sciences, University of Pretoria, South Africa; b Department of Biological Sciences, Faculty of Sciences, Eduardo Mondlane University, Maputo, Mozambique

**Keywords:** Children under five, Diarrhoea, Diagnosis, Treatment, HIV, Human Immunodeficiency Virus, INE, Instituto NAcional de Estatistica, ORS, Oral Rehydration Solution, SPSS, Statistical Package for the Social Sciences, WHO, World Health Organization

## Abstract

**Background:**

Although diagnosis and treatment of diarrhoea are considered easy, statistics show that 525,000 children worldwide die annually due to diarrhoea, 90% of the deaths are in Sub-Saharan Africa and South Asia, and Mozambique account for 6.9%. Assessment of practices of diagnosis and treatment of diarrhoea in children under five were con-ducted in Maputo, Mozambique.

**Design and method:**

The study was retrospective – source of information: record books from 2015 to 2019. All statements about age, gender, signs and symptoms, diagnoses, and treatment were collected to assess practices implemented by the health professionals to diagnose diarrhoea in children under five.

**Results:**

A total of 9,041 cases were found, where 4,052 (44, 8 %) were female, urban area accounts for 7,668 (74.8 %). Children younger than 6 months 1,013 (11,2%); from 6 to 11 months 1,370 (15,2%); from 12 to 23 months 2,535 (28 %); from 24 to 35 months 1,674 (18.5 %), from 36 to 47 months 1,239 (13.7 %) and from 48 to 59 months 1,210 (13.4 %). About 3,644 (40.3 %) had fever, 3,467 (38 %) vomit, 1,999 (22 %) blood in stool and other symptoms; only 5 (1 %) of the children’s stools were submitted for laboratory analysis. The clinical diagnoses were diarrhoea 3,905 (43 %), diarrhoea and vomit 2,037(22 %) and others. The main treatment was oral rehydration salts 7,118 (79 %) and 21 % antibiotics.

**Conclusion:**

Even when the signs and symptoms (fever and blood in stool) suggested or required laboratory exams, this was not done. Nevertheless, the children were treated with antibiotic without the screening of etiological agent.

## 1 Introduction

In 2017, the World Health Organization (WHO) reported nearly 1.7 billion cases of diarrhoea in children worldwide; the disease is the second most frequent cause of death in children younger than five (Abebe et al., 2018, Workie et al., 2019, Getachew et al., 2018). It amounts to one in eight deaths in children under five years of age (Kotloff, 2017). About 525,000 children continue to die each year because of the disease. Among the children who die each year 90 % happen to be in Sub-Saharan Africa and South Asia (Karambizi et al., 2021, Aziz et al., 2018). And diarrhoea is a common disease in children younger than five years of age in some communities of Mozambique (Nhampossa et al., 2015, Chissaque et al., 2018). Along the country, diarrhoeal diseases are considered to be the third leading cause of hospital admission in rural areas among children and the fourth cause of death among children aged 12–59 months (Nhampossa et al., 2015). According to WHO (2017) diarrhoea is defined as the loss of fluid or watery stools at least three times per day, or more frequently than normal (Levine et al, 2017); diarrhoea is also considered as a common symptom of gastrointestinal disorder caused by a wide range of pathogens, including parasites, bacteria, and viruses (Morais et al., 2013, Who, 2015).

Diagnosis of diarrhoea is centred on anamnesis, complete clinical examination, and adequate laboratory exam (Shane et al., 2017, Long et al., 2003, Guarino et al., 2018). Generally, the health providers use signs and symptoms to diagnose diarrhoeal diseases without confirmation of the laboratory results (Arendt et al., 2013). Signs and symptoms, such as the regularity and arrival of watery stools, incidences of vomiting, abdominal pain and related ailments, are used specifically to diagnose diarrhoeal diseases and to determine the treatment thereof. In addition, a physical examination is conducted and the observation of the skin turgor and elasticity is done. It is important to conduct laboratory examinations to assess and determine serum values of potassium, sodium, clorox, acid-base status, creatinine, glucose, biochemical limits of inflammation, normal urine analysis, and in certain cases, homo culture as well (Shane et al., 2017, Long et al., 2003, Farthing et al., 2013)Having laboratory results could confirm and assist the health provider in the treatment of diarrhoeal diseases and prevent complications, such as severe dehydration, loss of electrolytes and ultimately, death. A person with fever or bloody diarrhoea should be clinically evaluated and start on antibiotics treatment after confirmation of the agent.

The practice of pharmacologic treatments, such as antibiotics and antidiarrhoeal agents, is not recommended because the disease is normally self-limiting and their practice can be destructive for the organism of the children (Shane et al., 2017). Diarrhoeal diseases are treated with fluid therapy mostly oral rehydration. The solution is administered to treat diarrhoea by replacing the lost fluid and electrolytes, to give the body energy and to retain fluid. Health providers are advised to rehydrate children under the age of five immediately, using an oral rehydration solution to prevent increased chances of disability and death (Shane et al., 2017, WHO, 2004). According to WHO, in developing countries, half of diarrhoeal diseases are treated with antibiotics (De La Cabada Bauche et al., 2011, Belderok et al., 2011, Chakraborty et al., 2021). And most prescriptions of antibiotics are provided empirically without any previous screening of the agent (Chakraborty et al., 2021).

If diarrhoeal diseases are not treated properly, the children become malnourished, more vulnerable to diarrhoea and other infections and these increases their risk of death (Tickell et al., 2017).The question is, why is diarrhoea still being treated with antibiotics without laboratory screening. To address the question, retrospective data was collected and the information was used to assess how the health providers perform diagnoses and treatment of diarrhoea in children under five as the disease remains a health problem in this age group.

## 2 Methods

### 2.1 Study place

The study took place in four public referral health facilities of Maputo-Mozambique, one rural and three urban health facilities. The rural Health Centre is located in Marracuene District, Eastern part of Maputo City and provides primary health care services to around 12,000 people with several illnesses. Malaria is among the main diseases pointed out. However, many other health care services and programmes are offered, which include diarrhoeal diseases, sexual transmitted infections, antenatal care, family planning and non-communicable diseases (diabetes and high blood pressure) among other programmes and services. All three urban health facilities are in Maputo City, located in the extreme South of the country which occupies 346 km^2^, with 1,124,988 people, according to the National Institute of Statistics (INE Instituto Nacional de Estatística, 2020a, INE Imstituto Nacional de Estatística, 2020b). Mavalane General Hospital and Mavalane Health Centre are two hospitals on the same premises. The Mavalane Health Centre is part of 10 health centres which refer patients to Mavalane General Hospital and they are responsible for providing curative and preventive medical health assistance. The Central Hospital of Maputo is the major hospital of Mozambique. It receives patients from all parts of the country and offers all health services. Mozambique accounts for 58.124 health professionals and the Central Hospital of Maputo holds 3.431 (5.9 %). In the whole country the proportion health worker per 100.000 inhabitants is 109.3 (INE Instituto Nacional de Estatística, 2020a, INE Imstituto Nacional de Estatística, 2020b).

A quantitative method was used to assess the diagnosis and treatment of diarrhoeal diseases by the health providers (nurses, health technicians and doctors) at the selected health facilities in Maputo. The study used retrospective data available in the record books of the hospital from 2015 to 2019. Data was collected from the records of the health facilities in 2021, during 8 months (March to December) on working days from 8 AM to 2 PM. The collected data was categorized into five sections, namely, A-Biography, B-Main signals (signs) and symptoms, C-Laboratory examination, d-Treatment and E- Diagnosis. All records without information about age, gender, diagnosis and treatment were excluded. The study also excluded cases of diarrhoea determined by non-infectious origins, known or documented human immunodeficiency virus (HIV), and/or other immunocompromised status and known serious medical diseases.

### 2.2 Data collection

For the data collection, a check list was used and placed in Excel sheet to facilitate labelling of data during the collection. A team of three data collectors’ assistants entered the data on the computer. For quality control, piloting was done with about five instruments of each data collector's tools. The objective of piloting was to verify probable mistakes on the data collection tools, and familiarise research assistants with the instruments. Before the main study, all research assistants were trained by the principal investigator to ensure understanding of the instruments, criteria of inclusion and exclusion, as well as other aspects of data collection. Comments and suggestions identified from the pilot study were incorporated in the final tools and concerns from the research assistants were resolved. After the piloting, the developed instruments for data collection were evaluated for consistency and plausibility by the researcher and supervisors. Every-one in the research team (research assistants trained by the principal investigator) was aware of the objectives of the study and how the data should be collected to guarantee the success of the study.

### 2.3 Data analysis

The data was analysed using the Statistical Package for the Social Sciences (SPSS) software, version 23, and the Microsoft Excel database and the analysis was done with the support of a statistician. Descriptive statistics were performed to organise, interpret, and communicate the numeric information.

### 2.4 Ethical considerations

Ethical clearance was obtained from the Ethical Review Committee of the University of Pretoria. Permission was sought and obtained from the Central Hospital of Maputo, District Directorate for Women's Health and Social Action of Marracuene, and the Maputo City Health Directorate. The study started after clearance from the health authorities and all ethical commitments were observed and respected during the study.

## 3 Results

### 3.1 Socio-demographic characteristics

The present study assessed the diagnosis and treatment of diarrhoea in children under the age of five from 2015 to 2019. The records with missing information and damaged pages were not quantified. According to the results, 9,041 records had information about children with diarrhoeal diseases in the four health facilities of the study. Table 1 shows the cases of diarrhoea per health facility and the gender of children affected.

**Table 1:** Cases reported per health facility per area and gender.

Area	Health facility	Cases reported	% of cases reported	Cases (%)	Gender	
					Male	Female
Rural	Marracuene health centre	1,373	15,2	1,373 (25.2)	736 (8.1 %)	637 (7 %)
Urban	Mavalane health centre	1,209	13.4	7,668 (74.8)	680 (7.5 %)	729 (5, 9 %)
	Mavalane general hospital	3,004	33,2		1,533 (17 %)	1,471 (16.3)
	Maputo Central Hospital	3,455	38.2		2,040 (22 %)	1,415 (15,7)
Total		9,041	100	9,041	4,989 (55, 2 %)	4,052 (44, 8 %)

The Table 2 describes the cases of diarrhoea per age group. It can be seen that the cases concentrated in three age group (2, 3 and 4).

**Table 2:** Cases of diarrhoea reported per age group.

N	Age group (months)	No of reported cases	% of reported cases
1	<6	1,013	11.2
2	6–11	1,370	15.2
3	12–23	2,535	28.0
4	24–35	1,674	18.5
5	36–47	1,239	13.7
6	47–59	1,210	13.4
Total		9,041	100

### 3.2 Signs and symptoms and laboratory analyses

Table 3 shows the signs and symptoms found in the record books. Stools of only five (0, 1 %) children were submitted to laboratory analysis, whereas the majority 9, 036 (99, 9 %) was not submitted.

**Table 3:** Sign and symptoms of the children with diarrhoea.

Signs and symptoms	Reported		
	yes	No	Total
Vomit	3,467(38.3 %)	5,574 (61.7 %)	9,041 (100 %)
Fiver	3,644 (40.3 %)	5,397 (59.7)	9,041 (100 %)
Blood in stool	1,999 (22.1 %)	7,042 (77.9 %)	9,041 (100 %)
Combined signs and symptoms	9,038 (100 %)	3 (0, 01 %)	9,041 (100 %)

### 3.3 Treatment

For the treatment for diarrhoea drugs such as zinc sulphate, oral rehydration salts (ORS), metronidazole, cotrimoxazole and others not specified were used (Table 4).

**Table 4:** Drugs used to treat children with diarrhoea.

Drugs	Reported treatment		
	Yes	No	Total
Zinc Sulphate	5,399 (59.7 %)	3,642 (40.3 %)	9,041 (100 %)
ORS	7,118 (78.7 %)	1,923 (21.3 %)	9,041 (100 %)
Metronidazole	1,325 (14.7 %)	7,716 (85.3 %)	9,041 (100 %)
Cotrimoxazole	608 (6.7 %)	8,432 (93.3 %)	9,041 (100 %)
Antibiophilo	3,831 (42.4 %)	5210 (57.6 %)	9,041 (100 %)
Other Combined drugs	8179 (90.5 %)	862 (9.5 %)	9,041 (100 %)

### 3.4 Diagnosis

Table 5 describes the type of diagnosis reported in the record books. It can be observed that diarrhoea can occur alone or combined with other symptoms.

**Table 5:** Diagnosis reported in the record books.

Diagnose	Frequency	%
Diarrhoea	3905	43.2
Diarrhoea + Vomiting	2037	22.5
Diarrhoea + Cough	1680	18.6
Diarrhoea + Upper airway infections	557	6.2
Diarrhoea + Vomiting + Cough	103	1.1
Dysentery	225	2.5
Others	533	5.9
Total	9041	100

## 4 Discussion

According to the results of the present study, there seem to be more cases of diarrhoea in urban areas which were three times more than the cases reported in the rural areas. Comparing the two referral hospitals, Marracuene (Rural), and Mavalane General Hospital (urban), it can observed that cases in urban areas continue to be higher against rural. This finding could be due to the higher agglomeration of population in the urban regions, whereas the population in rural areas lives dispersed from each other. In addition, the users of Mavalane General Hospital mostly come from neighbourhoods with poor water and sanitation conditions. The findings of the present study could be supported by previous studies reporting the poorest sanitation conditions and hygiene in Maputo City (Mottelson, 2020), taking into consideration that Mavalane General Hospital, Mavalane Health Centre and Maputo Central Hospital are located in this city.

In this study, diarrhoea was higher among children from 6 to 35 months of age. This could be due the underdeveloped immune system of the children in addition the fact that in this age group children are starting to eat complementary food. The new foods could cause certain gastrointestinal disorders, which may result in diarrhoea. Furthermore, the hygiene quality of complementary food and water used for its preparation are other elements that could be taking into account, as poor hygiene of food and water could result in diarrhoea in children. It is well known that, in this age group, children begin to crawl then walk which result in more contact with the ground; where whatever they find they put in their mouths. Both the ground and objects could be contaminated by microorganisms that cause diarrhoea. Cases of diarrhoea in children under six months are not common. However, some cases were found in the record books, which could be due to early stopping of breastfeeding and early introduction of complementary food. The findings of this study are supported by previous researches which reported that in Mozambique diarrhoea infections in children under five particularly in age from 0 to 24 months are still a problem (Nhampossa et al., 2015, Chissaque et al., 2018, Chissaque et al., 2021). The cases of diarrhoea reported in the group from 36 to 59 months are considered normal, because the immunity system starts to consolidate and children start to be aware of the principles of hygiene such as how to wash hands and distinguish dirty and clean. A previous study reported similar findings (Gupta et al., 2015, Stephen et al., 2017).

Fever and vomiting were the main symptoms reported in children with diarrhoea. These symptoms could be the main cause of electrolyte disorder which consequently results in diarrhoea and if not treated in time, could end up in hypoglycaemia, convulsions and death. In the record books cases of blood in the stool were reported. When there is blood in the stool and fever, laboratory analysis to identify the agent is required. In this study, this recommendation was not followed, because only a few cases with blood in the stool were sent for laboratory exam. The practice reported was against the guideline of the Society of America Clinical Practices and WHO (Shane et al., 2017, WHO, 2004). This attitude of not screening for the agent even when the stool shows blood and the child had fever seems to be harmful as it can result in incorrect diagnoses.

Generally, the children were reported to be treated correctly as there were cases that were given oral rehydration salts and zinc sulphate which is in accordance with the universal guideline for diarrhoea treatment (WHO, 2004). Other types of treatment were given, but the use of antibiotics calls for attention. Following the procedures of looking for the diagnoses, antibiotics cannot be given without first knowing the agent. It can be considered that the procedures of treatment and diagnoses were not healthy as the study did not allow or determining whether the antibiotics were properly taken. All these together can result in antibiotic resistance. Previous studies have reported that empiric antimicrobial treatment is not recommended for diarrhoea (Shane et al., 2017, Mohanan et al., 2015). In Mozambique, antibiotic resistance have been reported (Vubil et al., 2018), as well as in other developing countries around the world (Mohanan et al., 2015, Mediratta et al., 2010, Carvajal-Velez et al., 2016, Wilson et al., 2012), what could be the arbitrary use of antibiotic.

Diarrhoea infection was common in the record books, and this was found alone or combined with other symptoms. The clinical diagnoses were reported as diarrhoea with vomiting, cough, dysentery and others. These diagnoses were found based on reported signs and symptoms without laboratory confirmation.

### 4.1 Limitations

Due to the covid-19 pandemic it was not possible to collect data in some health facilities previously selected for the study, some data was not found because of inappropriate conservation of the record books some data was not correctly filed and other was omitted there were books with missing pages. As way to surpass the constrains other health facilities were identified. Inappropriate conservation or missed information influenced the coverage of the research.

## 5 Conclusion

All in all, diarrhoea is diagnosed without screening for the etiological agent even in children with fever and blood in the stool. Treatment is done according to universal recommendations. However, some cases were treated with antibiotics without knowing the cause. It is advisable to create conditions to screen diarrhoeal agents especially in case of fever and blood in the stool.

## Declaration of Competing Interest

The authors declare that they have no known competing financial interests or personal relationships that could have appeared to influence the work reported in this paper.
